# Distribution of Electron Density in Self-Assembled One-Dimensional Chains of Si Atoms

**DOI:** 10.3390/ma16176044

**Published:** 2023-09-02

**Authors:** Mieczysław Jałochowski, Tomasz Kwapiński

**Affiliations:** Institute of Physics, Maria Curie-Sklodowska University, 20-031 Lublin, Poland; tomasz.kwapinski@mail.umcs.pl

**Keywords:** atomic chains, charge density waves, Friedel oscillations, electron occupancy along the chain, Si(553)-Au surface, STM, tight binding

## Abstract

Scanning tunneling microscopy measurements of height profiles, along the chains of Si atoms on the terrace edges of a perfectly ordered Si(553)-Au surface, reveal an STM bias-dependent mixed periodicity with periods of one, two and one and a half lattice constants. The simple linear chain model usually observed with STM cannot explain the unexpected fractional periodicity in the height profile. It was found that the edge Si chain stands for, in fact, a zigzag structure, which is composed of two neighboring rows of Si atoms and was detected in the STM experiments. Tight-binding calculations of the local density of states and charge occupancy along the chain explain the voltage-dependent modulations of the STM profiles and show that oscillation periods are determined mainly by the surface and STM tip Fermi energies.

## 1. Introduction

Among the many effects occurring in metallic one-dimensional (1D) atomic chains, charge density waves (CDW) [1,2,3,4] are frequently observed and can be easily investigated with a scanning tunneling microscope (STM). The concept of this effect assumes that metallic 1D chains are unstable at low temperatures, which was originally proposed by R. Peierls in 1955 [5]. The main mechanisms that are responsible for CDW in 1D systems are the collective displacement of atoms, electron–electron interactions, or electron–phonon couplings [4,6,7]. In a perfectly ordered system, CDW modulation is commensurate with the position of atoms, but any lattice imperfection, including 1D structure termination, generates CDW with characteristic length decay. Note, also, that spin-dependent CDW with spin-up and the spin-down electron waves are characterized by having no lattice distortion [8].

Another effect associated with the wave nature of electrons in 1D systems is known as Friedel oscillations (FO) [9] and it appears in regular systems with spatially disturbed potential (by lattice imperfections, impurities, and dislocations) or in confined atomic systems due to boundary effects [10,11,12]. This effect was theoretically predicted by J. Friedel, and it manifests as an oscillatory behavior of the electronic density around an impurity potential [13,14]. Friedel oscillations have first been investigated by means of the STM technique on cooper surfaces [15] where standing wave patterns near point defects and atomic steps were observed. This effect was also found in 1D electron gas and atomic chains [7,16] with a characteristic sinusoidal decay of the oscillation amplitude in the fermionic density near the perturbation. The rate of oscillation decay depends mainly on the system dimensionality [17] and may influence the stability and other properties of metallic atomic chains as well as of ultrathin metallic films [18]. The impurity-induced Friedel oscillations along the system allow one to get information about the electronic properties of the investigated system. For a multilayer graphene, the interference FO pattern reveals the band structure of this material, and allows to determine the quantized Berry phases as well as the number of stacked layers [19]. Note that FO and CDW can appear simultaneously in regular systems that are confined (by boundaries) or have charge impurities inside [20,21]. As a result, one should observe standing electron waves with higher intensity near the system imperfections and boundaries where charge waves are expected to be more evident.

To our best knowledge, there are no experimental results on the total electron occupancies along the chain. Such occupancies at each chain site can be derived theoretically and they often form a regular charge wave. Instead, in experiments, scientists show the STM height profiles or STM currents (with the topographic images) with well-visible oscillations and call them FO or CDW. In such a case, these waves that are formed along the chain should be almost independent of the STM tip and the applied STM voltage. However, in most experiments the waves show strong dependence on the STM parameters (they are voltage-dependent and, in particular, they can change the oscillation period) which is unclear and should be thoroughly explained.

In this paper, we experimentally and theoretically study the problem of charge waves in atomic chains on the surface. Both CDW and FO concern spatial variability of the local density of states (LDOS) and overlap in most cases, leading to standing charge waves along the system. It is known that the periodicity of these waves is determined by the system Fermi energy. However, for the chain between two electrodes out of equilibrium or in the STM geometry, there are two Fermi energies that influence the charge oscillation period. Thus, we predict that the net charge modulation along the chain should depend on both Fermi energies and can be a combination of two periods. This problem is also reflected in the interpretation of the STM results due to at least two reasons: firstly, for the constant current operation of the STM, the height profile carries data on both the topography and integrated LDOS modified by the tunneling transmission coefficient; secondly, a reliable interpretation can only be made for metallic systems where sample biases are close to zero and the bias energy window is small. For larger biases, the shape of the system LDOS in the bias window (between the surface and STM Fermi energies) should determine the chain occupancy and can significantly modify charge waves in the system leading to bias-dependent oscillation periods. In this work, we resolve this problem and unambiguously identify the nature of voltage-dependent periodicities using the experimental studies and theoretical tight-binding calculations.

In order to corroborate our predictions, we are going to investigate Si atomic chains on a Si(553)-Au surface by means of the STM topography method. Next, for a chosen finite chain, the detailed height profiles along the chain will be recorded. This allows us to answer the question of whether (and how) the oscillation structure of measured curves change with the positive and negative STM biases. In our theoretical calculations it is crucial to consider the atomic chain on a substrate which corresponds to the experimental setup and can be useful to explain the charge waves behavior along the chain and to verify which physical parameters are responsible for the observed effects. The atomic chain located at the edge of Si(553)-Au terraces seems appropriate for investigating the problems mentioned above. However, recent intensive experimental and theoretical investigations of Si atomic chains at the edge of Si(553)-Au terraces [22,23,24,25,26,27,28,29] assumed a single, double, triple, or even six-unit cell as a building block of terraces, including an atomic chain of Si atoms at the edge. These diversities were caused by the fragile atomic structure of the surface prone to transformation upon temperature variation [30], doping [31] caused by tunneling currents or adsorbtion of hydrogen atoms [32,33]. Note, however, that the origins of different Si chain periodicities on Au decorated Si(553) surfaces are still under discussions [23,30,34,35,36,37,38]. STM techniques are the methods of first choice for studying the atomic structure of atomic chains, therefore it seems necessary to thoroughly understand the charge distribution effects with integer and fractional periodicities. To our best knowledge, until now, the bias-dependent fractional periodicity of the STM height profiles of Si atomic chains on Si(553)-Au surface has not been reported in the literature. Our experimental and theoretical studies deliver a semi-quantitative explanation of the observed fractional periodicity in the height profile along the Si chain and the bias-dependent variation of the periodicity upon sample bias. Since all experimentally studied atomic chains on different surfaces interact with the substrate, the results of the presented work should be of more general importance.

## 2. Experiment: Methods and Results

### 2.1. Experimental Details

The experiments were carried out in an ultra-high vacuum system with a base pressure in the middle of the range of 10${}^{-11}$ mbar. The system was equipped with a Reflection High Electron Energy Diffraction (RHEED) diffractometer, OMICRON LT STM/AFM apparatus, gold deposition sources, and a precise quartz microbalance sensor. N-type Si (553) samples with a specific resistivity of 0.002 ÷ 0.01 Ω· cm were cleaned according to the standard procedure for silicon samples. After several hours of degassing, the samples were finally cleaned at a temperature of about 1500 K using DC flashing under RHEED control until a clean surface without SiC contamination was achieved. Next, 0.48±0.02 ML of Au (in units of one half of the Si(111) surface atom density equal to $7.84\times 10^{14}$ atoms/cm${}^{2}$) was deposited onto a substrate held at room temperature. The desired well-ordered surface, in the form of one-dimensional structures of various lengths running along the periodically arranged atomic steps, was obtained after heating the sample at 950 K for 2 s and gradual cooling to room temperature over 3 min. During resistive heating, the current was directed parallel to the steps. Scanning tunneling topography measurements in the constant current and constant height modes were carried out at 4.6 K and/or 77.4 K. All sample preparation steps were controlled with the RHEED diffractometer.

### 2.2. Atomic Structure of Si Edge Chains on Si(553)-Au

**Figure 1:**
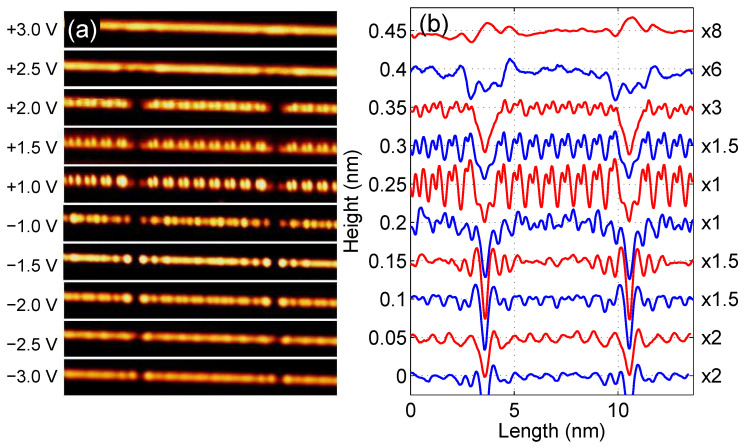
(**a**) A series of topographic images of the same Si edge atomic chain of Si(553)-Au sample. Left side labelling denotes sample bias. Images were recorded with tunneling current of 50 pA at 4.6 K. (**b**) Corresponding height profiles. Notice labelling to the right where signal multiplication factor is displayed. For better visibility, the curves are shifted successively by 0.05 nm and the colors are changed.

Figure 1 shows topographic images (panel a) and corresponding height profiles (panel b) for the edge Si atomic chain of the Si(553)-Au sample recorded with a tunneling current of 50 pA and for sample biases from −3 V up to +3 V at 4.6 K. In each profile curve, two minima can be seen due to defects defining the length of the atomic chain as equivalent to 17 × $a_{Si}$, where $a_{Si}=0.384$ nm is the length of the bulk Si lattice unit along the $\left[1\bar{1}0\right]$ direction. The nature of these defects has been previously investigated, and their origin was found to be due to the adsorption of residual water molecules [39]. We concentrate on finite length Si chain where FO and CWD effects should be more evident. A chain of this length (which consists of N = 35 Si atoms) will later be used as a model for theoretical calculations.

As one can see, the height profiles show different oscillation periods along the chain. While the −2.0 V, +1.0 V, +1.5 V and +2.0 V profile curves clearly show the presence of the $2\times a_{Si}$ modulation superimposed on the usual $1\times a_{Si}$ oscillations, the other profiles are more complicated, especially those for −1.5 V and −1.0 V showing modulation of $1\frac{1}{2}\times a_{Si}$. Moreover, curves with bias below −1.5 V show symmetrically decaying oscillations at both ends that are related to FO.

**Figure 2:**
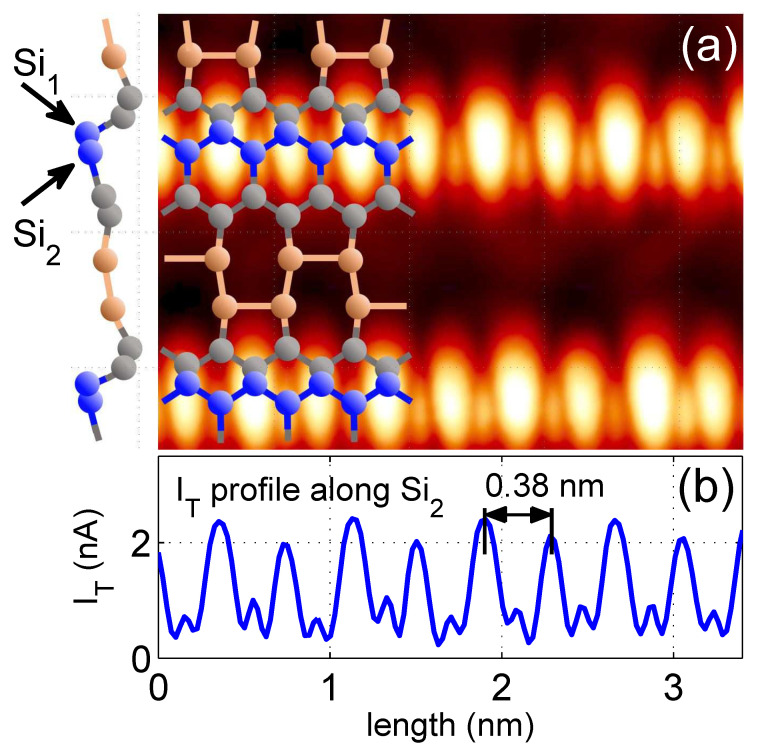
(**a**) a high-resolution constant-height image of a Si(553)-Au terrace, with structural model of Si(553)-Au surface atoms published in reference [25]. To the left, a side view of this model is displayed. $Si_{1}$ and $Si_{2}$ atoms form a zigzag chain discussed in the article. Panel (**b**) shows tunneling current profile along chain formed by $Si_{2}$ atoms. Large peaks originate from $Si_{1}$ atoms with unbound orbitals. The smaller ones come from $Si_{2}$ bound to four other Si atoms. The image was recorded at 77 K with a sample bias of +1.2 V and setting of a minimal STM loop gain. The distance between larger neighboring peaks corresponds to Si lattice unit cell length along the $\left[1\bar{1}0\right]$ direction.

Several theoretical works [25,26,28,30] indicate that Si atoms at the step edge of the Si(553)-Au surface belong to a honeycomb structure formed from other Si atoms on the terrace, as shown in more detail in Figure 2, where a high-resolution, constant-height image of a Si(553)-Au terrace with Si atoms at the step edge are displayed. Bright features are identified as edge atoms forming the $Si_{1}$ atomic chain. Together with the $Si_{2}$ atoms of the next neighbouring parallel row (see Figure 2a), they form a zigzag type chain (blue balls in the atomic scheme) where the distance between $Si_{1}$ (and between $Si_{2}$) atoms is 0.384 nm, while between $Si_{1}$ and $Si_{2}$ it is only 0.222 nm [25]. In STM topographic and in constant height images, atoms $Si_{1}$ dominate due to their unbounded orbitals. Therefore, in order to also resolve neighboring $Si_{2}$ atoms in the composed chain, the image was recorded with a relatively large current, reaching up to 2.4 nA and setting a minimal STM loop gain. The experiment was performed at 77 K with a sample bias of +1.2 V. Figure 2b shows the tunneling current profile along the line of $Si_{2}$ atoms. Smaller peaks clearly indicate that there are other Si atoms between the edge sites screened by $Si_{1}$ edge atom orbitals. Thus, our STM experiments confirm the existence of the Si edge chain, which is composed of two atomic rows in the zigzag geometry; this conclusion constitutes a very important result for this paper. In our theoretical mode we assume that the couplings between neighboring atoms ($Si_{1}-Si_{2}$) are defined by hopping integrals t and $t^{\prime }$ for interactions between ($Si_{1}-Si_{1}$), with the dominant role of t.

## 3. Theoretical Description

### 3.1. Model and Calculation Method

To describe the electronic properties of the considered chain at the surface, we use tight-binding calculations, which can successfully capture the essential physics of low-dimensional materials [40,41,42]. The system is modeled by a finite-length chain composed of *N* atomic sites in the zigzag configuration, as shown schematically in Figure 2 (blue balls), which corresponds to Si chains at the terrace edge of the Si(553)-Au surface. Such a system can be described by the following second quantization Hamiltonian:

(1) $$\begin{array}{ccc} H & = & \sum_{i=1}^{N}\varepsilon_{i}a_{i}^{†}a_{i}+\sum_{\vec{k}}\varepsilon_{\vec{k}}a_{\vec{k}}^{†}a_{\vec{k}}+\sum_{i=1}^{N}\sum_{\vec{k}}V_{\vec{k_{i}},i}a_{\vec{k}}^{†}a_{i}+\sum_{<i,j>}t_{i,j}a_{i}^{†}a_{j}+h.c. \end{array}$$

The operators $a_{i}^{†},a_{i}$, $a_{\vec{k}}^{†}$, $a_{\vec{k}}$ are creation/annihilation operators at the *i*-th atomic site, or in the surface electrode in the $\vec{k}$ state, respectively, and $\varepsilon_{\vec{k}}$ ($\varepsilon_{i}$) corresponds to possible electron energies in the surface (atomic chain). Here, the summation over *i* runs over all atomic sites in the chain, i=1,…,N, and the sum over <i,j> in the last term in Equation (1) includes both the neighboring and next-neighboring atomic sites. The parameter $V_{\vec{k},i}$ stands for the couplings (hybridization elements) between the states in the surface and in the chain.

It is worth noting that, in our model, electron–electron interactions are assumed to be irrelevant and can be captured by an effective shift of the chain’s onsite energies, such that they do not lead to correlation effects. Then, both spin directions are independent of each other, and spin indexes in the Hamiltonian are not written explicitly. Moreover, we consider only regular chains with the same hopping integrals between the neighboring atomic sites, $t_{i,j}=t$, and uniform on-site electron energies, $\varepsilon_{i}=\varepsilon_{0}$. The next-neighbor couplings between the step Si atoms can also be considered along the chain and, for them, we assume $t_{i,j}=t^{\prime }$. These terms can play a role mainly for positive STM voltage, as in this case the electron density near the surface increases, which leads to more occupied Si unbounded states with nonzero hybridization elements (hopping integrals), $t^{\prime }$.

To analyze charge distribution along the chain, we need to obtain the on-site electron occupancy from the formulae:

(2) $$\begin{array}{c} n_{i}=\int_{-\infty }^{E_{F}}LDOS_{i}\left(E\right)dE\;, \end{array}$$

where LDOS(E) is the local density of states function at a given *i*-th site and the zero temperature case is assumed. The LDOS function is obtained within the framework of the Green’s function method from the relation: $LDOS_{i}\left(E\right)=-\frac{1}{\pi }ImG_{ii}^{r}\left(E\right)$, where $G_{ii}^{r}\left(E\right)$ is the retarded Green function related to the *i*-th site of the chain. This function can be found from the knowledge of the Hamiltonian and the equation of motion technique [43]. After some algebra, one obtains the set of algebraic complex equations for $G_{ij}^{r}$, which can be written in the matrix form: $\hat{A}\;\cdot {\hat{G}}^{r}=\hat{I}$, where $\hat{I}$ is the unit matrix, and $\hat{A}$ matrix elements read:

(3) $$\begin{array}{c} A_{ij}=\left(E-\varepsilon_{0}\right)\delta_{ij}+i\frac{\Gamma_{ij}}{2}-t\delta_{i,j\pm 1}-t^{\prime }\delta_{i_{odd},j\pm 2} \end{array}$$

The spectral density function, $\Gamma_{ij}\left(E\right)=2\pi \sum_{k}V_{\vec{k}i}V_{\vec{k}j}^{*}\delta \left(E-\varepsilon_{\vec{k}}\right)$, stands for the effective chain–surface coupling and, in general, depends on the electron localization in the substrate. In our calculations, we model this function within a wideband approximation as energy independent, such that $\Gamma_{ij}\left(E\right)=\Gamma \delta_{ij}$.

Note that the $t^{\prime }$ parameter concerns every second atomic site, but only within the Si topmost edge chain ($Si_{1}-Si_{1})$, and the delta function includes only the odd *i* index, $\delta_{i_{odd},j\pm 2}$. Thus, by finding the inverse of $\hat{A}$ we can find the matrix of retarded Green functions $G^{r}$, i.e.,

(4) $$\begin{array}{c} G_{ij}^{r}\left(E\right)=\frac{\mathrm{cof}{\hat{A}}_{ij}}{\det\hat{A}}\;, \end{array}$$

where $\mathrm{cof}\hat{A}$ is the algebraic complement of $\hat{A}$ matrix (so-called cofactor). Diagonal elements of $G_{ij}^{r}\left(E\right)$ allow us to obtain the LDOS function and then the occupancy. For a regular chain on a substrate without the next neighboring couplings, $t^{\prime }=0$, the matrix $\hat{A}$ is tridiagonal and its determinant satisfies the recurrence relation and can be expressed analytically in terms of the Chebyshev polynomials of the second kind [44,45]. In our case, for this determinant, we use the product representation which allows us to write the $G_{ii}^{r}\left(E\right)$ function as:

(5) $$\begin{array}{c} G_{ii}^{r}\left(E\right)=\frac{\prod_{j=1}^{i-1}\left(E-\varepsilon_{0}+i\frac{\Gamma }{2}-2t\cos\frac{j\pi }{i}\right)\prod_{j=1}^{N-i}\left(E-\varepsilon_{0}+i\frac{\Gamma }{2}-2t\cos\frac{j\pi }{N-i+1}\right)}{\prod_{j=1}^{N}\left(E-\varepsilon_{0}+i\frac{\Gamma }{2}-2t\cos\frac{j\pi }{N+1}\right)}\;. \end{array}$$

Also, the LDOS function can be obtained analytically in this case and, e.g., for the first chain site, i=1, one finds:

(6) $$\begin{array}{c} LDOS_{1}\left(E\right)=\frac{\Gamma }{2\pi }\sum_{j=1}^{N}t^{2(j-1)}\frac{\prod_{j_{1}=1}^{N-j}\left[{\left(E-\varepsilon_{0}-2t\cos\frac{j_{1}\pi }{N-j+1}\right)}^{2}+{\left(\frac{\Gamma }{2}\right)}^{2}\right]}{\prod_{j_{1}=1}^{N}\left[{\left(E-\varepsilon_{0}-2t\cos\frac{j_{1}\pi }{N+1}\right)}^{2}+{\left(\frac{\Gamma }{2}\right)}^{2}\right]}\; \end{array}$$

The above analytical solution for the LDOS function allows us to analyze the positions of LDOS peaks in the energy scale. These peaks appear for minima of the determinant in Equation (6), i.e., for $E=\varepsilon_{0}+2t\cos\frac{j\pi }{N+1}$, where j=1,…,N is an integer number, and for *N*-site chain one expects *N* peaks in LDOS. Moreover, Equation (6) is used to obtain the electron occupancy from Equation (2), however, the transparent analytical form of $n_{i}$ in the general case is difficult to derive.

For atomic chains, the condition for the charge waves is closely related to the conductance oscillations effect [36,46,47]. Then, the charge oscillations with the period of *M*-sites along the chain occur if the following condition is satisfied:

(7) $$\begin{array}{c} \frac{E_{F}-\varepsilon_{0}}{2t}=\cos\frac{m\pi }{M}\; \end{array}$$

where m=1,…,M−1, and $E_{F}$ is the Fermi energy of the surface. For example, the oscillation with the period of M=3 sites along the chain can be observed for $E_{F}-\varepsilon_{0}=\pm 2t$ (here, sign+ is for m=1 and sign− for m=2). Similarly, the charge oscillations with the period of M=2, M=4, M=5 or M=6 appear for $E_{F}-\varepsilon_{0}=0$, $E_{F}-\varepsilon_{0}=\pm \sqrt{2}t$, $E_{F}-\varepsilon_{0}=\pm \sqrt{2}t$, $E_{F}-\varepsilon_{0}=\pm \frac{\sqrt{5}\pm 1}{2}t$, and $E_{F}-\varepsilon_{0}=\pm \sqrt{3}t$, respectively. A detailed discussion with graphical illustrations of these conditions are given in the next section. Note that charge oscillation period, *M*, is closely related to the periodicity of height profiles along the Si atoms, which is usually expressed in terms of the lattice constant, $a_{Si}$ (distance between every second site in the zigzag edge chain). In this case, experimentally observed periodicity (in $a_{Si}$ units) corresponds to the half periodicity of the zigzag chain, M/2.

### 3.2. Charge Oscillations

**Figure 3:**
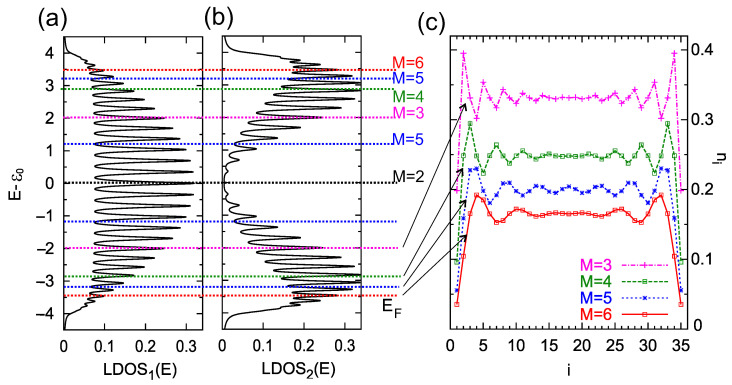
Local density of states at the first atomic site, $LDOS_{1}\left(E\right)$, (panel **a**), and the second site $LDOS_{2}\left(E\right)$, (panel **b**), for the chain length N=35 atoms and for t=2, $t^{\prime }=0$, Γ=0.1, $\varepsilon_{0}=0$. The dashed colorized lines indicate the positions of the Fermi energy for which charge oscillations along the chain occur with a period of M=2 (black line), M=3 (magenta lines), M=4 (green lines), M=5 (blue lines), and M=6 (red lines), respectively. (**c**) Charge occupancy, $n_{i}$, at every site of the chain, i=1,…,N, for the Fermi energy: $E_{F}=-t$ (which corresponds to the oscillation period M=3), $E_{F}=-\sqrt{2}t$ (M=4), $E_{F}=-t\left(\sqrt{5}+1\right)/2$ (M=5), and $E_{F}=-\sqrt{3}t$ (M=6), from the upper to bottom curves (as is depicted in the legend). The lines are a guide for the eye.

The relative position between the Fermi energy and the on-site electron energies in the chain determines the appearance of charge oscillations [36,46]. According to Equation (7), it is also important how strong the hopping integrals are between the neighboring sites, *t*. To illustrate the relation between the LDOS function and the charge oscillations in the chain, in Figure 3 we analyze the role of the surface Fermi energy on the charge oscillation period. In panel (a), the energy-dependent LDOS at the first atomic site, i=1, is shown for the chain length N=35 and the site–site coupling t=2. As one can see, this function is nonzero in the energy range (−2t,+2t), as is predicted for infinity 1D TB chains, and is characterized by *N* peaks, which appear for all minima of the determinant in Equation (6). Panel (b) shows the LDOS function for the second atomic location, i=2, with an overall shape different from the first, but with the same number of LDOS peaks. The different intensities of these LDOS peaks at both sites lead to different occupancies of these sites by electrons.

The horizontal colorized dashed lines in panels (a) and (b) indicate the energy positions of the surface Fermi energy for which the condition for charge oscillations is satisfied with the period of M=2 (black line, $E_{F}=0$), M=3 (magenta lines, $E_{F}=\pm 2$), M=4 (green lines, $E_{F}=\pm 2\sqrt{2}$), M=5 (blue lines, $E_{F}=\pm \left(\sqrt{5}\pm 1\right)$), and M=6 (red lines, $E_{F}=\pm 2\sqrt{3}$). These energies were calculated using Equation (7). Note that instead of changing the Fermi energy of the system, $E_{F}$, one can equivalently change the on-site energies of the chain, $\varepsilon_{0}$, to obtain the same oscillation periods (for fixed $E_{F}$).

In Figure 3, panel (c), electron occupancy along the chain is depicted for the chosen positions of $E_{F}$ which correspond to the charge oscillations with the period of M=3,4,5, and 6 (from upper to bottom curves, respectively). As can be seen, the electron occupancy along the chain varies depending on the Fermi energy of the substrate with very well visible periodicities. These oscillations are more intense near the ends of the chain, as expected for FO.

The effect of charge density waves in atomic systems is widely reported in the literature and is investigated mainly using the STM technique. The profile heights or the STM electron current often show intriguing oscillations in space but, in fact, these oscillations depend on the local DOS only between the surface and the STM chemical potentials, $E_{F}^{surf}$ and $E_{F}^{STM}$. This is the reason they cannot represent the total occupancy at a given site (which depends on LDOS below Fermi energy), but they are related only to the *biased window charge*, Δn. In this case, the STM current is directly proportional to this quantity:

(8) $$\begin{array}{c} I_{STM}\;\;∼\;\int_{E_{F}^{surf}}^{E_{F}^{STM}}LDOS_{i}\left(E\right)dE=\Delta n_{i}\;, \end{array}$$

Note that, for small biases (small difference between the STM and surface Fermi energies), the tunneling current is proportional to the LDOS function at the Fermi energy. For greater voltages, one expects a more complex dependence of the current as the LDOS function in the bias window varies from site to site. Thus, it is desirable to investigate how this function (bias window charge) changes along the chain for different voltages and whether it reveals the Friedel oscillation effect.

**Figure 4:**
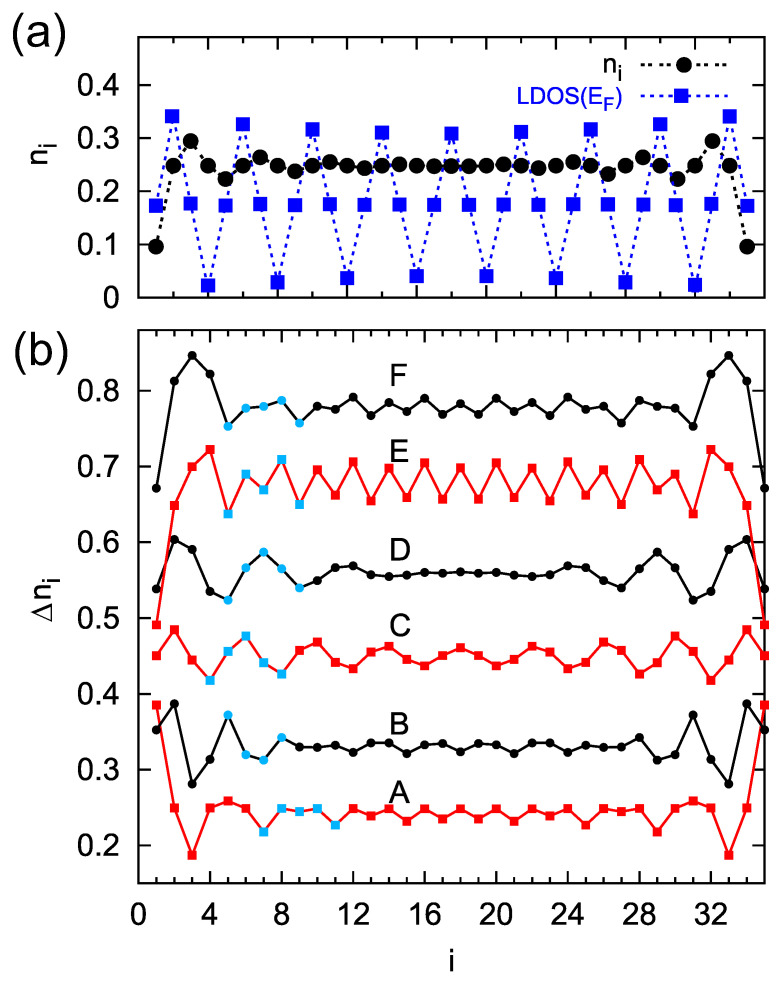
(**a**) Charge occupancy, $n_{i}$ (black dots), and the LDOS at the Fermi level (blue squares) along the chain composed of N=35 atomic sites and $E_{F}-\varepsilon_{0}=-\sqrt{2}t$ (the oscillation period is M=4), t=2, $t^{\prime }=0$. Panel (**b**) presents the bias window charge (LDOS integrated over the energy between the surface and STM Fermi energies), $\Delta n_{i}$, along the same chain for $E_{F}^{surf}=0$ and $E_{F}^{STM}=-2.75,-1.0,-0.2,+0.4,+0.7,+1.5$, curves from A to F, respectively. For negative $E_{F}^{STM}$: t=2, $t^{\prime }=0$, and for positive ones: t=2, $t^{\prime }=0.4$, and all B–F curves are shifted for better visualizations. The characteristic periodic structures at each curve in (**b**) are indicated by the blue marks.

In the beginning, we analyze the total charge (obtained according to Equation (2)) along the chain composed of N=35 sites, where the condition for the period of M=4 sites is satisfied, $\varepsilon_{0}=+\sqrt{2}t$ (Figure 4, panel (a), black dots). The Friedel oscillations are very visible, with decreasing oscillation amplitude towards the chain center. Additionally, in this panel, the LDOS function at the surface Fermi level, $LDOS(E_{F})$, is depicted (blue squares), which also shows oscillations with a period of four sites. However, in contrast to the Friedel oscillations, this function oscillates with a constant amplitude and does not decrease along the chain. Thus, for a small voltages, the STM current oscillations can be observed along the whole atomic chain, $I_{STM}\;\;∼\;\;LDOS\left(E_{F}\right)$ but, in addition to the same oscillation period, it does not duplicate the Friedel oscillations in the system.

The situation is more complicated for larger STM voltages where the current depends on the bias window charge, Δn, which we study in Figure 4, panel (b). The curves represent Δn distribution along the chain for negative and positive voltages, i.e., for the STM Fermi energy $E_{F}^{STM}=-2.75$ (curve A) up to +1.5 (curve F). Now, for each curve, the surface Fermi level corresponds to the oscillation period of four sites ($\varepsilon_{0}=+\sqrt{2}t$), whereas the position of the STM Fermi energy can satisfy the condition for other charge oscillation periods, thus the four-site oscillations of Δn are modified in such a case. For example, curve A is a composite of M=4 and M=2 periods, giving the net four-site oscillations. The position of the STM Fermi energy for curve B corresponds to the oscillations with a period of three sites, and these oscillations dominate in the system. Note that curve C is obtained for a small bias voltage, and its structure is similar to the oscillations of the LDOS function (panel a). Also, for possitive STM Fermi energies, the curves reveal bias-dependent structures. Thus, by changing the STM voltage, one can register various shapes and different periods of the STM current along the chain. This important result shows that both surface and STM tip Fermi energies determine the charge oscillation period. The system parameters in our numerical calculations were chosen in order to satisfy qualitative agreement with the experimental data. In the calculations, all the energies are expressed in units of $\Gamma^{0}=1$, such that for $\Gamma^{0}=0.2$ eV the surface coupling strength is below 1 eV and the site–site coupling is t=0.4 eV.

**Figure 5:**
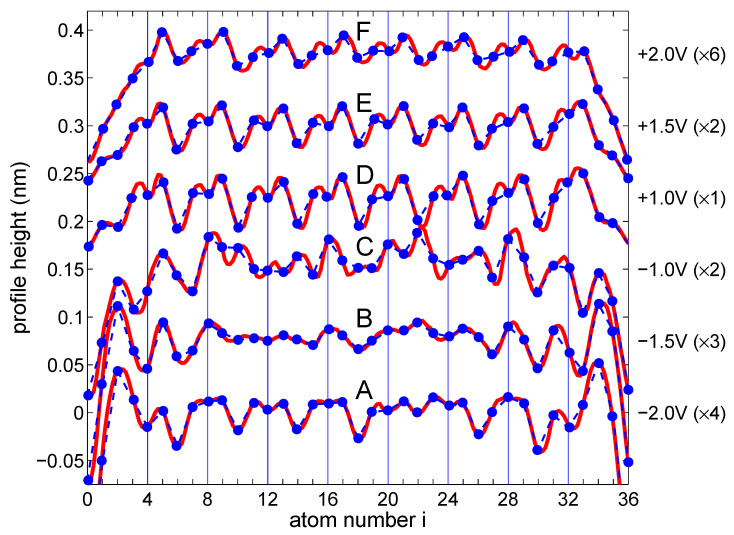
Selected parts of experimental height profile (red lines) as shown in Figure 1 with marked positions of Si atomic chains considered in the theoretical calculation. The blue dots and dashed lines have been drawn for easier comparison with the theoretical $\Delta n_{i}$ (bias window charge) profiles presented in Figure 4. The right side label indicates the bias voltages and the signal multiplication factors for each curve. For better visibility, the curves are shifted successively by 0.075 nm from the bottom. Note the periodicity of $1\frac{1}{2}\times a_{Si}$, $2\times a_{Si}$ and the superposition of those depending on applied biases.

To corroborate our theoretical findings with the experimental results, it is necessary to analyze in more detail the height profiles for the Si zigzag chain depicted in Figure 1 (right panel). In Figure 5, such selected experimental curves (red lines) with marked positions of the Si atoms (blue dots) that form a zigzag chain are depicted. Evidently, in height profiles measured at biases of −2.0 V, +1.0 V, +1.5 V, and +2.0 V an oscillation period of $2\times a_{Si}$ dominates (which corresponds to the period of M=4 atoms in the zigzag chain), whereas, on profiles recorded at −1.5 V, we observe the period of $1\frac{1}{2}\times a_{Si}$ (which is equivalent to M=3), and for −1.0 V the oscillation periodicity of $2\times a_{Si}$ mixed with $1\frac{1}{2}\times a_{Si}$ oscillations are seen. One should note that, despite the same periodicity of some curves in Figure 5, their shapes differ from each other, especially for positive and negative voltages.

To compare these experimental height profiles with the theoretical results shown in Figure 4, panel (b), there are highlighted parts (blue dots in Figure 4) in each A–F curve and, as one can see, they have quite similar/equivalent structures to the curves shown in Figure 5 (the corresponding curves are arranged from the bottom to the top at both figures and are labelled by the letters A–F). It shows that the STM profiles observed in the experiment along the Si chain are related to the bias window charge. Here, one can notice that not only general shapes of the theoretical and experimental curves are consistent, but also the oscillation periods match each other, e.g., for the oscillation period M=3 (curve B in Figure 4), the step Si chain reveals the period $1\frac{1}{2}\times a_{Si}$ (curve B for −1.5 V in Figure 5), and this is similar for other curves. Thus, it can be seen that the height profile curves do not simply represent the total charge oscillations, which stand for the Friedel oscillations, but the $\Delta n_{i}$ quantity (bias window charge). Although the theoretical results are not fully in line with the experimental ones due to the relatively simple model of the TB chain, they address the main electronic features of the system and explain the bias-dependent nature of STM topography oscillations in 1D systems.

## 4. Conclusions

In this work, we analyzed the STM height profiles along one-dimensional Si atomic chains located at the edges of the Si(553)-Au surface terraces. We found that STM height profiles vary with integer and fractional 1D lattice periods, which was not expected for a simple monoatomic chain. Similar fractional periodicity of tunneling current was seen in tunneling current profiles along the Si step-edge chain. We also observed that the STM profile curves change their shape and periodicity depending on the applied voltages. As a consequence, the edge chain cannot be considered as a simple linear row of atoms but it has a structure of a zigzag geometry. The proposed setup of this chain is composed of a row of edge Si atoms and, not detected in previous STM experiments, a nearest row of Si atoms on the terrace. This conclusion constitutes the main result of the paper.

To explain the bias-dependent oscillations in the STM experiments, the tight binding model Hamiltonian of an atomic chain was considered, and the local density of states and charge occupancy were obtained. For a regular chain on a surface, analytical solutions for the retarded Green function and LDOS were derived. As further important results of the work, it was found that: (i) the profiles of the heights or currents along the chain (which are related to the bias window charge) depend mainly on the Fermi energies of both the substrate and the STM tip, which determine the resultant oscillation periods in the system; and (ii) charge occupancies along the chain change their distribution and oscillation period for different STM voltages. This semi-quantitatively explains the voltage-dependent relation of the STM profiles. Note that all experimentally studied atomic chains considered in this paper are very stable and regular; additionally, they interact with the substrate, thus the results of the presented work should be of more general importance for 1D atomic structures.

## Data Availability

Data are available from the authors upon reasonable request.
